# PLK1 inhibitors as a new targeted treatment for adrenocortical carcinoma

**DOI:** 10.1530/EC-23-0403

**Published:** 2023-12-14

**Authors:** Emily Warmington, Gabrielle Smith, Vasileios Chortis, Raimunde Liang, Juliane Lippert, Sonja Steinhauer, Laura-Sophie Landwehr, Constanze Hantel, Katja Kiseljak-Vassiliades, Margaret E Wierman, Barbara Altieri, Paul A Foster, Cristina L Ronchi

**Affiliations:** 1 Institute of Metabolism and System Research, University of Birmingham, Birmingham, UK; 2 Division of Endocrinology and Diabetes, University Hospital of Wuerzburg, Wuerzburg, Germany; 3 Department of Neurosurgery, Technical University Munich (TMU), Munich, Germany; 4 Department of Endocrinology, Diabetology and Clinical Nutrition, University Hospital Zurich (USZ) and University of Zurich (UZH), Zurich, Switzerland; 5 Medizinische Klinik Und Poliklinik III, University Hospital Carl Gustav Carus, Dresden, Germany; 6 Division of Endocrinology Metabolism and Diabetes, University of Colorado Anschutz Medical Campus, Aurora, Colorado, USA; 7 Centre for Endocrinology, Diabetes and Metabolism, Birmingham Health Partners, Birmingham, UK

**Keywords:** adrenocortical carcinoma, polo-like kinase 1, molecular-targeted therapy

## Abstract

Adrenocortical carcinoma (ACC) is an aggressive malignancy with limited treatment options. Polo-like kinase 1 (PLK1) is a promising drug target; PLK1 inhibitors (PLK1i) have been investigated in solid cancers and are more effective in *TP53*-mutated cases. We evaluated PLK1 expression in ACC samples and the efficacy of two PLK1i in ACC cell lines with different genetic backgrounds. PLK1 protein expression was investigated by immunohistochemistry in tissue samples and correlated with clinical data. The efficacy of rigosertib (RGS), targeting RAS/PI3K, CDKs and PLKs, and poloxin (Pol), specifically targeting the PLK1 polo-box domain, was tested in *TP53*-mutated NCI-H295R, MUC-1, and CU-ACC2 cells and in *TP53* wild-type CU-ACC1. Effects on proliferation, apoptosis, and viability were determined. PLK1 immunostaining was stronger in *TP53-*mutated ACC samples vs wild-type (*P* = 0.0017). High PLK1 expression together with *TP53* mutations correlated with shorter progression-free survival (*P*= 0.041). NCI-H295R showed a time- and dose-dependent reduction in proliferation with both PLK1i (*P*< 0.05at 100 nM RGS and 30 µM Pol). In MUC-1, a less pronounced decrease was observed (*P*< 0.05at 1000 nM RGS and 100 µM Pol). 100 nM RGS increased apoptosis in NCI-H295R (*P*< 0.001), with no effect on MUC-1. CU-ACC2 apoptosis was induced only at high concentrations (*P* < 0.05 at 3000 nM RGS and 100 µM Pol), while proliferation decreased at 1000 nM RGS and 30 µM Pol. CU-ACC1 proliferation reduced, and apoptosis increased, only at 100 µM Pol. *TP53*-mutated ACC cell lines demonstrated better response to PLK1i than wild-type CU-ACC1. These data suggest PLK1i may be a promising targeted treatment of a subset of ACC patients, pre-selected according to tumour genetic signature.

## Introduction

Adrenocortical carcinoma (ACC) is a rare yet highly aggressive endocrine malignancy with generally poor prognosis (1). Treatment options for ACC are scarce, with the only potential curative therapy being complete resection (2). However, post-surgical recurrence rate is high and associated with dismal clinical outcomes. The adrenolytic mitotane is the only approved drug for treatment of patients with advanced disease (3), while cytotoxic chemotherapies such as etoposide–doxorubicin–cisplatin (EDP) and gemcitabine plus capecitabine represent alternative options, but all show low response rates and frequent adverse effects (4, 5). Although our understanding of ACC’s heterogeneous pathogenesis has improved through pan-genomic molecular studies, targeted therapies are not yet available. Previous molecular screenings provided some promising insight into potential pharmacological targets (6, 7, 8, 9) and the efficacy of available inhibitors was investigated in small clinical studies. Nevertheless, results have been largely unsatisfactory (reviewed in (10, 11)). Linsitinib, a dual inhibitor of the insulin-like growth factor 1 receptor (IGF1R) and insulin receptor (IR), is the only targeted drug to have entered a phase III trial for ACC patients (OSI-906) but also yielded disappointing results (12).

**Figure 1:**
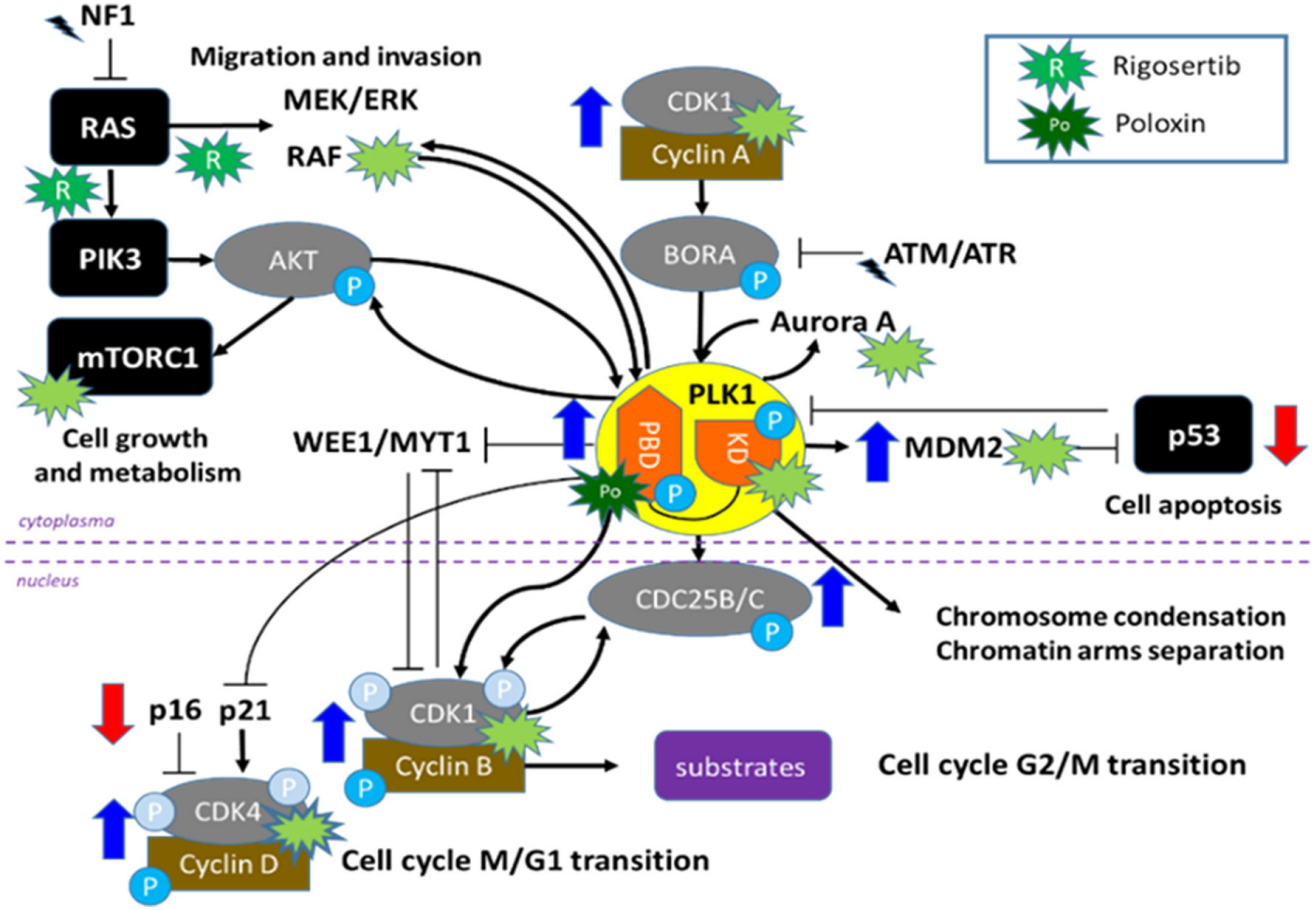
Schematic representation of the interplay between Polo-Like Kinase 1 (PLK1) and other cell cycle-related pathways, such as CDK families (i.e. CDK1 and CDK4), p53, RAS/PIK3, and mTOR. The inhibitory effects of the two investigated PLK1 inhibitors (multi-targeting rigosertib, RGS, and PBD-PLK1 specific poloxin, Pol) are highlighted.

In a recent study, we performed targeted gene expression profiling of ACC tumour samples and identified up-regulated genes and pathways, including cyclin-dependent kinase (CDK) and polo-like kinase (PLK) families (9), whose inhibition may represent promising treatment options. In particular, PLK1 is an important regulator of mitotic entry and progression, involved in the feedback loop that activates CDK1 by promoting CDC25 activation. PLK1 also inhibits p53-dependent transcriptional activation and pro-apoptotic activity, and, in turn, p53 represses *PLK1* expression itself (Fig. 1) (13). Overexpression of *PLK1* at gene level has been reported to be associated with worse clinical outcome, as shown in a previous study that merged available expression data from microarray studies (14) with those reported by Demeure *et al.* (15). Of note, also in the TCGA cohort, *PLK1* overexpression was significantly associated with unfavourable outcome (16, 17).

PLK1 is highly expressed in many solid malignancies; hence, several PLK1 inhibitors (PLK1i) have been evaluated in clinical trials (18, 19, 20, 21, 22, 23, 24, 25, 26). These PLK1i included first-generation BI-2536, second-generation ATP-competitive BI-6727 (volasertib), and non-ATP-competitive ON 01910.Na (rigosertib), targeting the RAS/PI3K pathway and CDKs besides PLK. Interestingly, PLK1 inhibition seems to be more effective in *TP53*-mutated tumours (16, 27, 28, 29). Recently, promising new-generation PLK1i specifically targeting the PLK1 polo-box domain (PBD), which is important for subcellular localisation, molecular mediation, and targeting of PLK activity towards specific subcellular domains (i.e. bringing the kinase domain in proximity with its substrates), have been tested in preclinical studies (26).

In ACC, *PLK1* has been shown to be frequently overexpressed (9, 16, 17) and associated with shorter patient survival (16, 17). Moreover, the first-generation inhibitor BI-2536 has been demonstrated to reduce cell viability and induce apoptosis in standard ACC cell lines (NCI-H295R and SW13).

The aim of this study was to test the potential role of targeting PLK1 for individualised treatment of patients with advanced ACC. To this end, we investigated the relationship between PLK1 expression and clinical outcome in a large cohort of well-characterised ACC tissue samples and evaluated the efficacy of two PLK1i on four ACC cell lines with different genetic backgrounds.

## Materials and methods

### Analysis of *PLK1* mRNA expression in existing datasets

We first re-evaluated *PLK1* gene expression levels in three previously published ACC data sets, including i) series from Giordano and colleagues (14) (*n* = 65 snap-frozen samples, i.e. 10 normal adrenal glands (NAG), 22 adrenocortical adenomas (ACA) and 33 ACCs investigated by microarrays), ii) series from The Cancer Genome Atlas (TCGA) cohort (30) (*n* = 79 snap-frozen samples examined by whole transcriptome RNA-sequencing, RNA-seq) and iii) our published series of 40 formalin-fixed paraffin-embedded (FFPE) samples investigated by targeted gene expression profile (9). We focused on the relationship between expression levels of *PLK1* and other cell cycle-related genes (i.e. CDKs, RAS, PI3K, topoisomerase, etc.).

### Patient cohort and clinical data

A total of 104 patients with histologically confirmed ACC, available targeted DNA sequencing data (8) and FFPE tumour specimens from whole tissue blocks, collected between 2002 and 2016, were included. Baseline clinical and histopathological characteristics (i.e. sex, age, adrenal hormone pattern, initial European Network for the Study of Adrenal Tumors (ENSAT) tumour stage, resection status of primary tumour, Ki67 proliferation index), as well as follow-up information, survival data and details about pharmacological treatment (i.e. mitotane and/or cytotoxic chemotherapies) were collected through the ENSAT registry (https://registry.ensat.org//) and patients’ records. These details are summarised in Table 1.

**Table 1:** Demographic, clinical, and histopathological characteristics of the 104 patients with adrenocortical carcinoma evaluated for PLK1 immunohistochemistry.

Parameter	Value
**Demographic and clinical parameters**	
Sex (M/F)	45/59
Age – years (median, range)	49 (18–87)
Initial ENSAT tumour stage (*n*)	
1–2	56
3	27
4	22
Pre-operative steroid secretion (*n*)	
Cortisol	23
Other single steroids (androgens, mineralocorticoids, or oestrogens)	9
Mixed steroids	21
Inactive	25
**Histopathological parameters**	
Ki67 index – % (median, range)	15 (1–90)
Resection status (*n*)	
R0	73
RX	16
R1	5
R2	8
Unknown	3
Tumour localisation (*n*)	
Primary surgery	86
Local recurrence	8
Distant metastasis	10
**Post-surgical pharmacological treatment**	
Adjuvant mitotane (*n*)	38
Palliative mitotane (*n*)	38
Cytotoxic chemotherapy	63

ENSAT, European Network for the Study of Adrenal Tumors; F, female; M, male; *n*, number of patients; R0, complete resection; R1, microscopic incomplete resection; R2, macroscopic incomplete resection; RX, uncertain resection.

The clinical outcome of patients with ACC was assessed by overall survival (OS) and progression-free survival (PFS) (see statistical analysis for definitions).

The study protocol was approved by the local ethics committee (University Hospital of Wuerzburg, #88/11) and written informed consent was obtained from all subjects prior to study enrolment.

### Immunohistochemistry

Protein expression levels of PLK1 in ACC samples and their relationship with genetic background, clinical/histopathological parameters and clinical outcome were evaluated. Immunohistochemistry (IHC) was performed on standard full sections of 104 ACC specimens and 11 benign ACAs. A total of five NAGs were used as negative controls. After deparaffinisation, antigen retrieval was achieved by heating the slides for 13min in the pressure cooker in 10 mM citric acid monohydrate buffer (pH 6.5). Unspecific binding sites were blocked with 20% human AB serum at room temperature (RT) for 1h and slides were then incubated at RT for 1h with specific antibodies against PLK1 (anti-mouse monoclonal PLK1 antibody 13E18 by ThermoFisher: dilution 1:50) or N-Universal Negative Control anti-mouse (Dako, Golstrup, Denmark). Antibody binding was detected by means of the En-Vision System Labelled Polymer-HRP and developed for 10min with DAB Substrate Kit (Vector Laboratories, Burlingame, CA, USA). Nuclei were counterstained with Mayer’s haematoxylin.

Evaluation of stained slides was performed by two independent operators blinded to the results and clinical information (R.L. and S.St.) using the Scope A1 microscope (Carl Zeiss AG). Intensity of nuclear staining and percentage of positive cells was graded as 0 (negative), 1 (low), 2 (medium), and 3 (high). The proportion of positive tumour cells was calculated for each slide and scored 0 if 0% were positive, 0.1 if 1% to 9% were positive, 0.5 if 10% to 49% were positive, and 1 if ≥ 50% were positive. A semi-quantitative *H*-score was then calculated by multiplying the staining intensity grading score with the proportion score (31, 32). In case of discrepancies, slides were jointly assessed by both investigators and a final score was developed by consensus. The Spearman’s correlation for interobserver agreement for each staining was high (*r* > 0.85). Representative examples of nuclear weak and strong PLK1 staining are shown in Supplementary Fig. 1 (see the section on supplementary materials given at the end of this article).

### ACC cell lines and culturing

We evaluated the potential anticancer activity of PLK1i in four different ACC cell line models. These included the standard ACC cell line NCI-H295R (33) and more recently developed MUC-1 (34), CU-ACC1 and CU-ACC2 cells (35). NCI-H295R cells were cultured in Dulbecco’s modified eagle medium (DMEM)/F12, HEPES media) (Gibco, 11330032), supplemented with 2.5% Nu-Serum growth media supplement (Corning, 355100), 1% insulin, human transferrin, and selenous acid (ITS) Premix (Corning, 354352) and 1% penicillin–streptomycin (Pen-Strep) (Gibco, 15070063). NCI-H295R were authenticated by Short Tandem Repeat (STR) analysis. Their doubling time is 25 h. MUC-1 cells were cultured with Advanced DMEM/F12 media (Gibco, 12634010) supplemented with 10% heat-inactivated fetal bovine serum (FBS) (Gibco, 10500064), and 1% Pen-Strep (34). Their doubling time is 60 h (36). CU-ACC1 and CU-ACC2 cells were cultured in media consisting of three parts F-12 Nutrient Mixture (Gibco, 11765054) to one part DMEM high glucose, pyruvate (Gibco, 11995065), supplemented with 10% FBS, 0.8% hydrocortisone (Sigma, H0888), 0.1% insulin (Sigma, I6634), 0.05% adenine (Sigma, A2786), 0.01% epidermal growth factor (Gibco, PHG0311), and 0.0084% cholera toxin (Sigma, C9903) (35). Their doubling time is 35 h and 29 h, respectively (35). Cell passages were comprised between 14 and 42.

### Molecular characterisation of ACC cell lines

Sequencing data for the four cell lines available from the literature demonstrate that they differ in genetic background (9, 34, 35). In particular, NCI-H295R carries a *TP53* deletion in addition to a *CTNNB1* activating missense mutation and an *RB1* loss, MUC-1 a frameshift *TP53* and *MEN1* mutation, CU-ACC2 a missense *TP53* mutation and *MSH2* deletion and CU-ACC1 as the only *TP53* wild-type cell line (with *CTNNB1* activating missense mutation). Additionally, we used a targeted gene expression profile containing 84 known drug targets (Cancer Drug Targets RT2 profiles, Qiagen) (9), to investigate the expression of drug targetable cell cycle-related genes in all four cell lines. We isolated RNA using the Maxwell RSC simplyRNA Tissue Kit (Promega) according to manufacturer’s instructions. Samples were transcribed with the RT2 First Strand Kit (Qiagen) according to the manufacturer’s protocol. Expression of a panel of 84 drug targetable genes as well as five housekeeping genes (ACTB, B2M, GAPDH, HPRT1, RPLP0) and seven positive control genes was evaluated by the Human Cancer Drug Targets RT2 Profiler PCR Array (PAHS-507Z, Qiagen). The reaction was performed with the RT2 SYBR Green qPCR Master Mix (Qiagen) and all cell lines were run in triplicate. Cycling conditions were 95^◦^C for 10min followed by 40 cycles of 95^◦^C for 15 s, 60^◦^C for 1 min. Fold change (FC) was calculated with the 2∧(-∆∆CT) formula normalised to five housekeeping genes and with a pool of five NAG from snap-frozen specimens as reference by the Qiagen GeneGlobe Data Analysis Center (https://geneglobe.qiagen.com/de). An FC of ≥2.0 was defined as high expression and an FC of ≥10.0 was defined as very high. The genetic and molecular characterisation of the four cell lines is shown in the Supplementary Fig. 2.

### Anticancer activity of PLK1 inhibitors

We evaluated the potential anti-cancer role of two different PLK1i: multi-targeting rigosertib (RGS) and new-generation PBD-specific poloxin (Pol). In order to test the efficacy of the drugs in a dose-dependent manner, increasing drug concentrations were used. Experiments were run for 72 h and results compared to a vehicle control consisting of media and DMSO. Based on review of previous literature, the following drug concentrations were selected for use in this project: 10, 30, 100, 300, 1000, and 3000 nM for RGS, and 1, 3, 10, 30, and 100 μM for Pol (37).

Cell proliferation was analysed using CyQUANT® Cell Proliferation Assay (Thermofisher, C7026), which quantifies cell proliferation using fluorescence-based techniques. Cell proliferation (reported as fluorescence relative to baseline) was measured after 72 h addition of PLKi to the cell and compared to control values for vehicle-treated cells. Rates of cell apoptosis were measured by Caspase-Glo® 3/7 Assay (Promega, G8091) after 72 h exposure to each PLKi, which detects caspase activity via luminescent signalling. CellTiter-Glo® Luminescent Cell Viability Assay (Promega, G7570) was used to assess cell viability after 72 h exposure to each PLKi using luminescence-based techniques corresponding to the amount of ATP present, a marker of metabolically active, hence viable, cells.

### Statistical analysis

Fisher’s exact or chi-square test was used to investigate dichotomic variables, while a two-sided *t*-test or non-parametric Mann–Whitney test was used to compare two groups of continuous variables as appropriate. A non-parametric Kruskal–Wallis test followed by Bonferroni *post hoc* test, was used for comparison among several groups for non-normally distributed variables. Correlations and 95% confidence intervals (95% CI) between different parameters were evaluated by linear regression analysis. For the data analysis related to the TCGA ACC dataset, RNASeq files (illuminahiseq_rnaseqv2-RSEM_genes_normalized) were downloaded from Firebrowse.org. Clinical data files (ACC merged_clinical) were also downloaded from the same source. Raw data for RNA-Seq was normalised by Log2 transformation and correlation curves generated. OS was defined as the time from the date of primary surgery to specific death or last follow-up, while PFS was defined as the time from the date of complete tumour resection to the first radiological evidence of disease relapse, progress or disease-related death. Survival curves were obtained by Kaplan–Meier estimates and the differences between two or more curves were investigated by the log-rank (Mantel–Cox) test. A multivariate regression analysis, including parameters with *P*-values below 0.1 at univariate analysis, was performed by Cox proportional hazard regression model to identify factors that might independently influence survival.

For cell line results a one-way ANOVA followed by a Tukey’s post-test was performed to compare data to relevant vehicle treated controls. Data was normally distributed as confirmed via the Kolmogorov–Smirnov normality test. All statistical analysis was performed with GraphPad Prism software 9.0 (GraphPad Software Inc.) or SPSS software (IBM SPSS statistics, version 29). *P*-values below 0.05 were considered statistically significant.

## Results

### *PLK1* gene expression in ACC samples (literature datasets)

**Figure 2:**
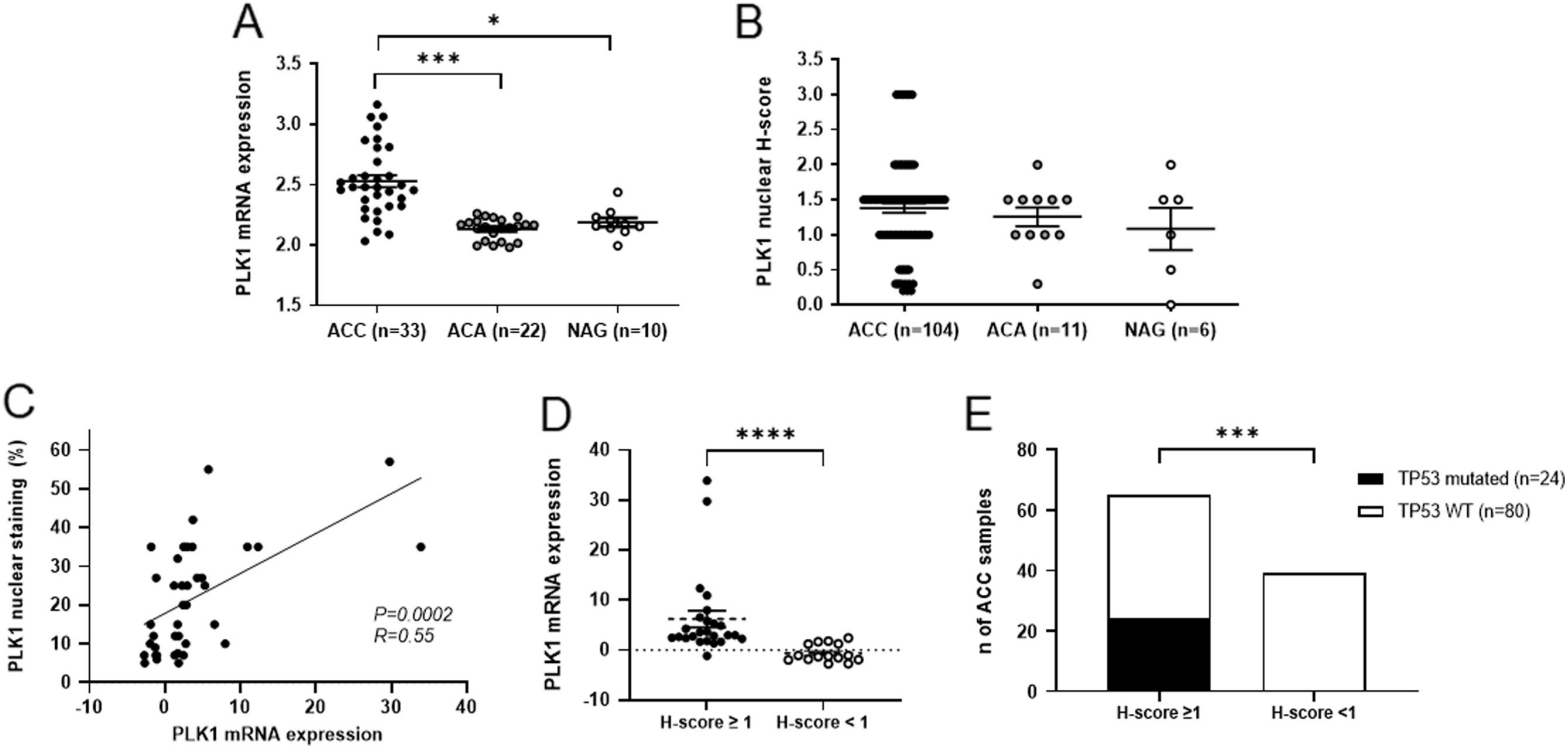
PLK1 gene and protein expression in adrenal tumour samples. (A) *PLK1* gene expression in a dataset of 33 adrenocortical carcinomas (ACCs), 22 adenomas (ACAs), and 10 normal adrenal glands (NAGs) from Giordano *et al.* (14). (B) Nuclear PLK1 staining evaluated by *H*-score in our cohort of 104 ACCs, 11 adrenocortical adenomas (ACAs), and 6 NAGs. *P* for trend = 0.697. (C) Relationship between PLK1 protein expression (percentage of positive nuclei) and gene expression levels in our cohort of 40 ACC samples. Statistics by linear regression analysis. (D) Relationship between PLK1 protein expression (*H*-score) and *PLK1* gene expression levels in our cohort of 40 ACC samples. ****P< 0.0005. (E) Relationship between PLK1 protein expression (*H*-score) and presence of somatic mutations in *TP53* gene (*n* = 104 ACC samples). ***P< 0.001.

In the transcriptome dataset from Giordano *et al.* (14), *PLK1* expression levels were higher in ACC than in both NAG and ACA (Fig. 2A, *P* < 0.005). Moreover, in our previously published cohort of 40 FFPE ACC samples (Liang *et al.*, 2020), *PLK1* mRNA levels were significantly correlated with several known anti-cancer drug targets, i.e. negatively with *AKT2*,* BIRC5*,* CDC25A, CDK2*,* CDK5*,* CDK7*,* CDK8*,* ESR1*,* FLT1*, and *GRB2* and positively with* HDCA1*,* HDCA2*,* HDCA4*,* HRAS*,* KIT*,* NFKB1PIK*,* PARP1*,* PIK3C2A*,* PLK4*,* TOP2A*,* TOP2B*,and *TXN.* The strongest and/or most biologically relevant correlations are shown in Supplementary Fig. 3. To further confirm these findings, we looked for the most significant correlations also in the TCGA RNA-seq dataset. Here, *PLK1* expression also positively correlated with *CDK8, CDC25A, PLK4*, and *TOP2A* (Supplementary Fig. 4), suggesting that these four gene targets may be of interest when considering *PLK1* inhibition in ACC.

### PLK1 protein expression in adrenocortical tumour FFPE samples

In our cohort of 104 FFPE ACC samples, the median percentage of cells with positive nuclear staining was 30% (ranging from 5 to 80%) while the median *H*-score was 1.5. PLK1 nuclear immunostaining was present in 84.6% of cases (*H*-score ≥ 0.2) and considered high (i.e. *H*-score ≥ 1) in 60%. There was no significant difference in nuclear staining intensity or percentage of positive cells among primary tumours, local recurrences or distant metastasis. When compared to ACA and NAG, PLK1 staining showed a trend of increased expression in ACC, even if this did not reach statistical significance (Fig. 2B). Furthermore, PLK1 protein expression positively correlated with mRNA expression levels (*n* = 40 ACC samples) for both percentage of positive cells (*P*< 0.001, *R* = 0.55, Fig. 2C) and *H*-score levels (*P*< 0.001,Fig. 2D).

For all 104 ACC cases, targeted DNA sequencing was available from a previous publication (8). Of note, PLK1 protein expression levels were higher in cases with somatic mutations affecting the *TP53* gene (*n* = 24) compared to wild-type tumours (*n* = 80) (*P*= 0.0045 by Mann–Whitney test) and all samples with *TP53* variants presented positive PLK1 nuclear staining compared to 80% of *TP53*-WT (*H*-score ≥1, *P*< 0.0001 by chi-square test, Fig. 2E).

### PLK1 protein expression in ACC samples and association with clinical outcome

We did not observe any significant correlation between PLK1 protein levels and clinical or histopathological parameters, including initial ENSAT tumour stage, steroid secretion pattern and Ki67 proliferation index.

**Figure 3:**
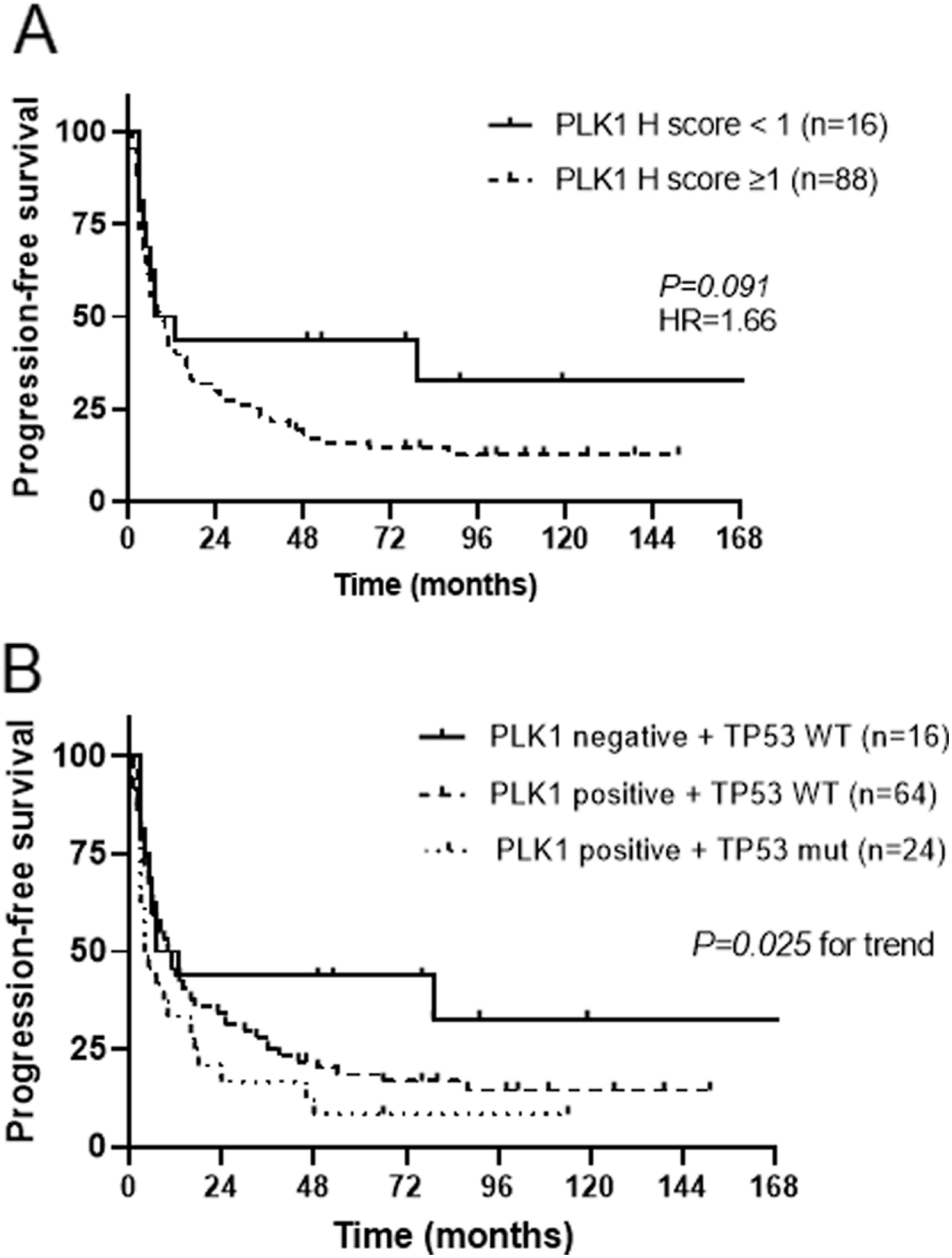
Relationship between PLK1 protein expression and clinical outcome evaluated as progression-free survival (PFS) in 104 adrenocortical carcinomas (ACC). (A) Kaplan–Meier curves for PLK1 protein expression (positive if *H*-score ≥ 1). (B) Kaplan–Meier curves for PLK1 protein expression and *TP53* gene mutations (WT, wild type). Statistical analysis by log-rank test.

Looking at the clinical outcome, there was a trend to a shorter PFS in patients with positive PLK1 nuclear staining (median survival 7.5 vs 17 months, *P*= 0.091, HR 1.66, 95% CI 0.98–2.83, Fig. 3A); however, this was not confirmed for OS (*P*= 0.89, HR 1.05, 95% CI 0.53–2.06, data not shown). Interestingly, patients with both positive PLK1 protein expression and somatic *TP53* mutations (*n* = 24) had a significantly shorter PFS compared to those *TP53-*WT with high PLK1 (*n* = 64) or low/absent PLK1 expression (*n* = 16) (median survival 4.5 vs 10.5 vs 10 months, *P*= 0.025 by log-rank test for trend, Fig. 3B). However, at multivariable analysis including clinical and pathological parameters, only ENSAT tumour stage and resection status remained significantly associated with PFS (*P*= 0.004, HR 1.60, 95%CI 1.16–2.21; and *P*= 0.036, HR 1.48, 95% CI 1.03–2.14 by Cox regression analysis), while ENSAT tumour stage and combined TP53 status-PLK1 expression showed only a trend (*P*= 0.087, HR 1.33, 95% CI 0.96–1.85; and *P*= 0.170, HR 1.30, 95% CI 0.89–1.88).

### Molecular characterisation of ACC cell lines

The genetic background of all four ACC cell lines is known from available literature (34, 35) (Supplementary Fig. 2). We characterised the gene expression of known anti-cancer drug targets using the same methods used for ACC tissue samples (Supplementary Fig. 2). Within cell cycle-related genes*, BIRC5,CDC25A,CDK1, PLK4,* and* TOP2A* were the homogenously highest expressed across all cell lines, followed by *CDK2* and *PLK1*. In particular, CU-ACC1 cells presented the highest expression (9.23-fold) of *PLK1*, while this was lower in MUC-1 cells (2.21-fold). A similar pattern was reflected with *IGF2* expression, though in this case, expression was very high in CU-ACC1 (136.08-fold), while it was under-expressed in MUC-1 cells (0.17-fold). Of note, CU-ACC1 cells presented some differences compared to other cell lines, i.e. higher expression of *CDK8, CDK9*, and *TERT* and a lower expression of *PLK2*.

### Effects of PLK1 inhibitors on ACC cell lines

**Figure 4:**
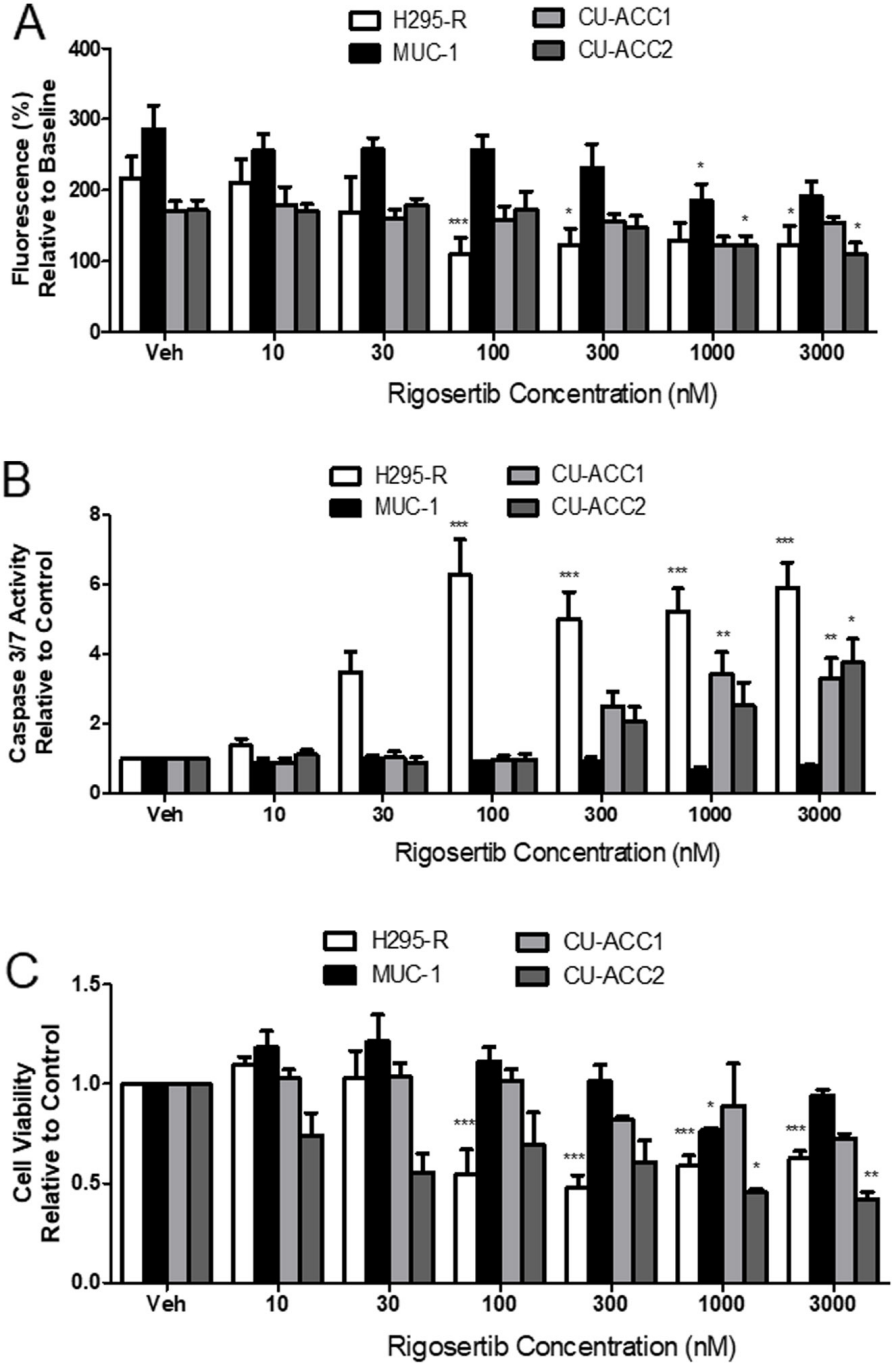
The effect of RGS on (A) cell proliferation, (B) caspase 3/7 activity and (C) cell viability in NCI-H295R, MUC-1, CU-ACC1, and CU-ACC2. Data represent a range of doses of RGS treatment after 72 h, *n* = 3–5 ± s.d. Statistical analysis is a one-way ANOVA followed by a Tukey’s post-test. *P < 0.05, **P > 0.01, ***P < 0.001 compared to the cell lines vehicle control (Veh).

As our data as well as previous literature suggest PLK1 may play a pathogenic role in ACC, we examined the ability of two PLK1i (multi-targeting RGS and PBD-specific Pol) to block ACC cell proliferation and survival. RGS reduced NCI-H295R proliferation by 50% (*P* < 0.001), 44% (*P* < 0.05), and 43% (*P* < 0.05) at 100, 300, and 3000 nM (*P* < 0.001), respectively, after 72 h treatment (Fig. 4A). RGS also caused an increase in caspase3/7 activity in NCI-H295R cells (*P* < 0.001) (Fig. 4B). At 100 nM, 300 nM, 1000 nM, and 3000 nM, RGS caused a 6.7-, 6.3-, 5.4-, and 5.7-fold increase in caspase 3/7 activity respectively. Furthermore, NCI-H295R cell viability was significantly reduced with doses above 100 nM RGS treatment (*P* < 0.001) (Fig. 4C).

In MUC-1 cells, RGS had much less impact on proliferation and cell viability. At the high doses of 1000 nM and 3000 nM, RGS lowered MUC-1 proliferation by 17.0% and 19.5%, respectively, after 72 h (*P* < 0.05), although this was only a modest slowing of cell growth compared to control (Fig. 4A). Indeed, when apoptosis and viability were examined in these cells, RGS was not effective at increasing caspase 3/7 activity (Fig. 4B) or lowering cell viability, other than a slight decrease at 1000 nM (Fig. 4C).

We further tested RGS in the more recently established CU-ACC1 and CU-ACC2 cell lines. In CU-ACC1, RGS slowed proliferation at 1000 nM; however, this reduction was not significant (Fig. 4A). At the higher doses of 1000 nM and 3000 nM, RGS caused a 3.4- and 3.3-fold increase in caspase 3/7 activity (*P* < 0.01), with a clear trend towards an increase at 300 nM (Fig. 4B). Viability of CU-ACC cells mimicked the proliferation data, with RGS having no significant effect on viability after 72 h treatment; however, there was a trend towards decreased cell viability in these studies (Fig. 4C). The effects of RGS in CU-ACC2 cells were more promising than in CU-ACC1. RGS reduced cell proliferation by 17.7% (*P* < 0.05) and 19.5% (*P* < 0.05) at 1000 nM and 3000 nM, respectively (Fig. 4A). This is supported by apoptosis results, which show that 3000 nM RGS increased caspase 3/7 activity by 3.8-fold compared to control (*P* < 0.01) (Fig. 4B). RGS at 1000 nM and 3000 nM also significantly lowered CU-ACC2 cell viability (*P* < 0.05 and *P* < 0.01, respectively) (Fig. 4C).

**Figure 5:**
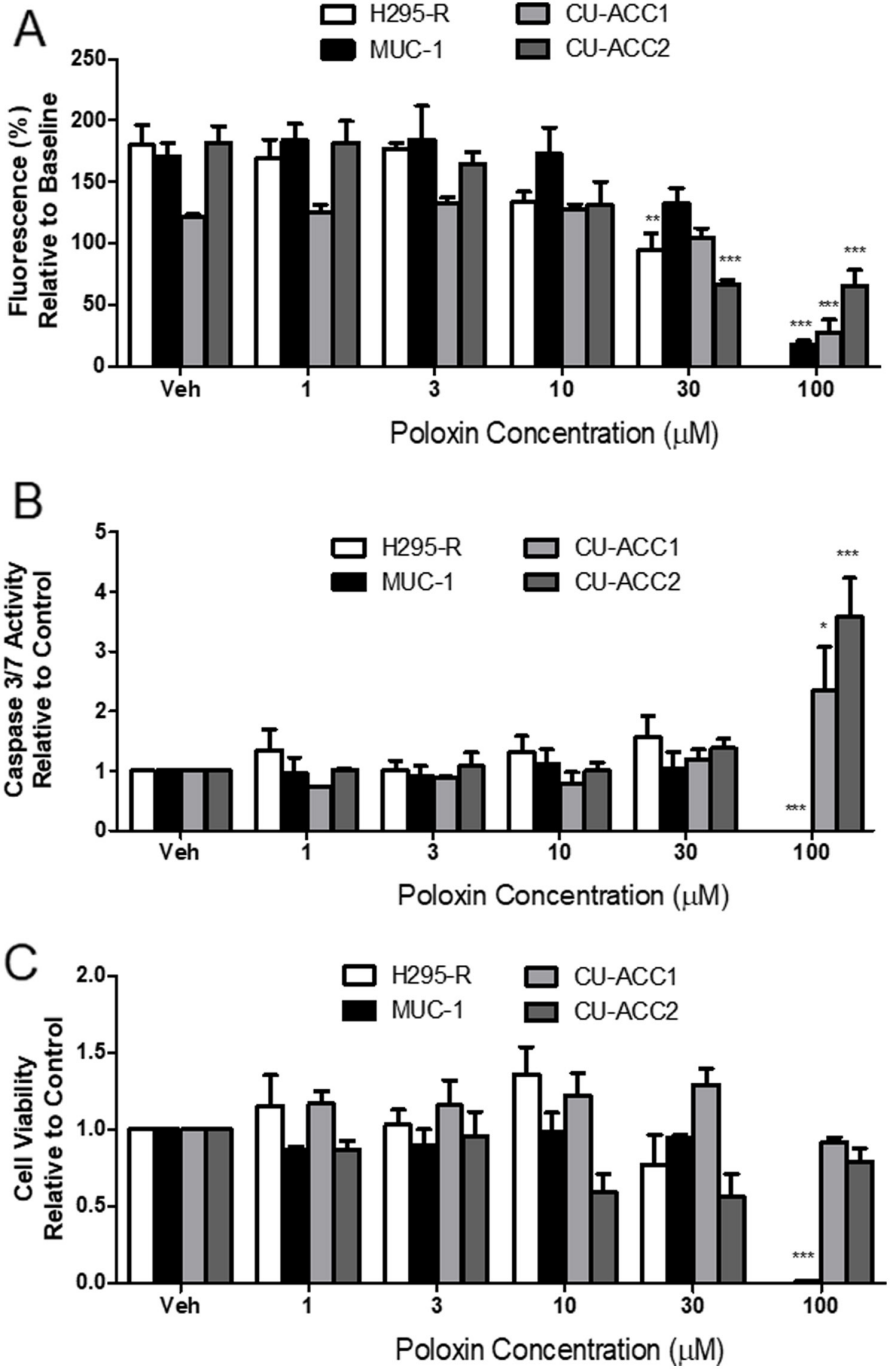
The effect of Pol on (A) cell proliferation, (B) caspase 3/7 activity and (C) cell viability in NCI-H295R, MUC-1, CU-ACC1, and CU-ACC2 cells. Data represent a range of doses of Pol treatment after 72 h, *n* = 3–4 ± s.d. Statistical analysis is a one-way ANOVA followed by a Tukey’s post-test. *P <0.05, **P >0.01, ***P <0.001 compared to the cell lines vehicle control (Veh).

Next, we tested the PLK1-specific inhibitor Pol on the same group of ACC cell lines. In NCI-H295R cells, Pol, at the high dose of 30 μM, caused a significant 48.8% reduction in proliferation over 72 h (*P* < 0.01) (Fig. 5A). However, at all doses tested, Pol did not affect caspase 3/7 activity (Fig. 5B) or NCI-H295R cell viability (Fig. 5C). In MUC-1 cells, 100 μM Pol treatment showed a 91.3% reduction in cell proliferation (*P* <0.001) (Fig. 5A). Caspase 3/7 activity was also entirely reduced by 100 μM Pol (*P* < 0.001) (Fig. 5B). MUC-1 cell viability was completely lost by 100 μM Pol treatment (*P* < 0.001) (Fig. 5C).

In CU-ACC-1 cells, Pol reduced proliferation by 77.9% only at the highest dose of 100 μM (*P* < 0.001); all other doses had no effect (Fig. 5A). When caspase 3/7 was examined, Pol caused a modest but significant 2.3-fold increase in activity at 100 μM only (*P* < 0.05), with all other doses showing no effect (Fig. 5B). Despite these results, no effect was seen on CU-ACC-1 cell viability at any dose (Fig. 5C). In CU-ACC-2 cells, Pol slowed proliferation by 63.6% and 64.0% at 30 μM and 100 μM, respectively (*P* < 0.001) (Fig. 5A). Caspase 3/7 activity was increased by 3.6 fold after 100 μM Pol treatment compared to control (*P* < 0.001) (Fig. 5B). However, no dose of Pol affected viability of CU-ACC-2 cells (Fig. 5C).

## Discussion

In this study, we have demonstrated that PLK1 represents a potential treatment target in ACC. Our findings might be of clinical relevance, given that ACC remains an aggressive malignancy with an urgent unmet need for molecular-targeted pharmacological therapies.

PLK1 represents an ideal anti-cancer drug target considering its role in mitotic regulation, interplay with the Rb/p53 pathway (38) and its overexpression in multiple solid tumours (26). Moreover, multiple PLK1i, including first-, second-, and third-generation drugs, have been investigated *in vitro*, *in vivo* and in clinical trials in other cancer types (reviewed in (13, 39)). Of note, it has been demonstrated that PLK1i are more efficacious in tumours harbouring variants in the *TP53* gene (16, 27, 28, 29, 40, 41). This is of interest, given that *TP53* mutations are present in approximately 30% of sporadic ACC cases (8, 30).

Concerning ACC, high *PLK1* mRNA expression has been reported in multiple studies (9, 16, 17, 30) and clearly linked to worse clinical outcomes (16, 17).

In this study, we investigated for the first time PLK1 expression at the protein level with immunohistochemistry in a large cohort of 104 ACC samples, showing that PLK1 is highly expressed in 60% of cases. We did not observe any significant relationship between PLK1 staining and clinical parameters or survival data. However, similar to many previous studies, our analysis is limited by its retrospective nature, as well as the potential influence of multiple systemic and local treatments after initial surgery. Importantly, we found a more evident trend to a poor prognosis when considering* TP53* mutation status in conjunction with PLK1 expression levels. Specifically, patients with both *TP53* mutations and high PLK1 expression had the shortest progression-free survival.

PLK1 is a potent oncogene and, therefore, an ideal drug target for anti-cancer therapy (42). In the present study, we investigated the efficacy of two types of PLK1 inhibition (multi-targeting RGS and small molecule PBD-specific Pol) in multiple ACC cell models. RGS was most effective against NCI-H295R cell growth and viability, but also had significant effects against CU-ACC2 cell proliferation and viability, and triggered increased apoptosis. It is unclear why RGS had less impact on MUC-1 and almost no effect on CU-ACC1 cells. It is possible that observed effects relate to RGS’s multi-targeting properties, i.e. inhibition of PLK1, CDK, and Ras (43). However, the CDK1/2 and KRAS expression profiles of MUC-1 and CU-ACC1 are similar to NCI-H295R and CU-ACC2 cells (see Supplementary Fig. 2), suggesting CDK is not underlying the difference in response to RGS’s effects. Instead, the fact that β-catenin is phosphorylated by Nek2 and subsequently by PLK1, and may itself be phosphorylated by PLK1 directly, may explain why NCI-H295R was more sensitive to PLK1 inhibition than other cell lines (44).

In clinical trials, despite its favourable pharmacokinetic profile, RGS showed limited success due to poor specificity, resulting in dose-limiting toxicity (45, 46). Therefore, PLK1i with stronger potency and higher selectivity have been developed and are currently under investigation in early phase trials (42, 47), i.e. third-generation PLK1i PCM-075 (onvansertib) and PLK1 siRNA TKM-080301 (21, 48) (https://clinicaltrials.gov/). Moreover, small-molecule PBD-specific PLK1i, such as Pol, have emerged as a novel, alternative class of inhibitors demonstrating proof of concept of *in vivo* efficacy (47). Therefore, to compare with the multi-targeting effects of RGS, we also tested Pol’s effects.

Pol was effective at blocking cell growth in all cell lines when tested at the highest dose of 100 μM. Interestingly, and similarly to our RGS findings, Pol also significantly impacted proliferation of NCI-H295R and CU-ACC2 cells at the slightly lower dose of 30 μM. When considering the molecular profiles of our ACC cell lines, again our results may suggest that PLK1 inhibition is more effective in ACC cells with specific *TP53* variants. In fact, NCI-H295R and CU-ACC2 cells harbour a *TP53* deletion or missense mutation, respectively, and were more sensitive to Pol treatment. MUC-1 cells also have a *TP53* deletion, however their PLK1 expression is less than in H295R and CU-ACC2, which may explain Pol’s reduced effect in them. In addition, the results observed in MUC-1 cells treated with high Pol concentrations (i.e. undetectable caspase 3/7 activity, below that of the normal MUC-1 turnover rate) may be explained by the cells entering a quiescent state, rather than dying.

CU-ACC1 cells were the least responsive to both RGS and Pol treatment. This cell line is *TP53* wild-type and, in our hands, were the slowest growing cells. This may be reflective of the fact that CU-ACC1 cells are not as reliant on the PLK1 pathway for early trigger of the G2/M transition. Further work is needed to examine which compounds or combinations could be effective at targeting non-*TP53* mutated ACC.

Overall, our cell data suggests that targeting PLK1 may be an effective treatment in a subset of patients with ACC. In fact, cell lines harbouring *TP53* variants demonstrated greater response to PLK1i than *TP53* wild-type CU-ACC1, with the most impressive efficacy being recorded in NCI-H295R cells. It is noted that, in our experiments, high doses of both RGS and Pol were used. Considering that maximum plasma RGS concentrations in published clinical trials are reported in the range of 0.20–5.93 μg/mL (29, 49), and clinically achievable concentrations of Pol are not yet known, replicating the dosages presented in this study *in vivo* may not be attainable. Nevertheless, our data are a proof-of-concept study, which suggest a potential role for PLK1 inhibition as a therapeutic target for ACC and provide a starting point for the development or identification of more efficacious compounds targeting PLK1. While not definitively providing evidence for use of Pol, we suggest more potent PLK1 inhibitors may be used against ACC in the future. Moreover, further studies on potential combination of PLK1i and other drugs targeting related pathways (i.e. CDK, mTOR or p53) are required. A depiction of known interplays between PLK1 and other potential additional drug targetable pathways and genes is shown in Fig. 1.

## Conclusion

In conclusion, we demonstrate that new-generation PLK1 inhibitors are effective in a subgroup of ACC cell lines with a specific genetic background. Therefore, we propose PLK1i as a promising targeted treatment of a subset of ACC patients that may be pre-selected according to their tumour’s molecular signature.

## Supplementary Materials

Suppl. Figure 1. Examples of representative nuclear PLK1 immunostaining. A) – B) Adrenocortical carcinoma (ACC) with weak PLK1 staining; C) Adrenocortical carcinoma (ACC) with strong PLK1 staining; D) Normal adrenal gland used as negative control. Magnification 20x. H-score calculated as described in Methods. Images taken with AxioCam MRm, Carl Zeiss AG, Jena, Germany.

Suppl. Figure 2. Schematic summarising molecular alterations observed in the four investigated ACC cell lines (NCI-H295R, MUC-1, CU-ACC1 and CU-ACC2). These include the response to treatment with PLK1 inhibitors rigosertib and poloxin (classified according to effects on cell proliferation), DNA alterations (i.e. single nucleotide variations and indels) and mRNA expression of cell cycle-related genes (investigated by RT-qPCR profile and reported as fold changes, see methods for details). A fold change of ≥2.0 was defined as high expression (light green), while a fold change of ≥10.0 was defined as very high expression (dark green). Fold change <0.50 was defined as low expression (red).

Suppl. Figure 3. Relationship of mRNA expression levels between PLK1 and other known anti-cancer drug targets. Data taken from our previously published cohort of 40 paraffin-embedded ACC samples (9). Shown are expression levels for significant correlations, i.e. negatively with AKT2, BIRC5, CDC25A, CDK2, CDK5, CDK7, CDK8, ESR1, FLT1, GRB2, and positively HDCA1, HDCA2, HDCA4, HRAS, KIT, NFKB1PIK, PARP1, PIK3C2A, PLK4, TOP2A, TOP2B, and TXN.

Suppl. Figure 4. Most significant correlations with PLK1 gene expression in our previously published dataset of 40 paraffin-embedded ACC samples (9) were analysed in the TCGA ACC RNAseq dataset. Of note, PLK1 expression also positively correlated with CDK8, CDC25A, PLK4, and TOP2A.

## Declaration of interest

We declare that there is no conflict of interest that could be perceived as prejudicing the impartiality of the research reported. Paul Foster is a senior editor of *Endocrine Connections*. Paul Foster was not involved in the review or editorial process for this paper, on which he is listed as an author.

## Funding

This work has been supported by the Deutsche Forschungsgemeinschafthttp://dx.doi.org/10.13039/501100001659 (DFG) within the CRC/Transregio (project number: 314061271 – TRR 205) and project RO-5435/3-1 (CLR), the Deutsche Krebshilfehttp://dx.doi.org/10.13039/501100005972 (project number 70112969 to CLR), the Graduate School of Life Sciences University Hospital of Wuerzburg (RL), and the AMEND ACC Research Fund 2021 (CLR). Moreover, this project has been carried out with the help of the Interdisciplinary Bank of Biomaterials and Data of the University Hospital of Wuerzburg and the Julius Maximilian University of Würzburg (IBDhttp://dx.doi.org/10.13039/100017412W) supported by the Federal Ministry for Education and Research (Grant number FKZ: 01EY1102). VC received support from the Academy of Medical Scienceshttp://dx.doi.org/10.13039/501100000691 UK (Starter Grant for Clinical Lecturers SGL020/1018). This work was additionally supported by Veterans Affairs Merit Review Award 001 and the Adrenal Tumor Program Fund (MEW), NIH K12CA086913-12 and Cancer League of Coloradohttp://dx.doi.org/10.13039/100009141 Award (KKV).
