# Supplementary figures and images for: PLK1 inhibitors as a new targeted treatment for adrenocortical carcinoma
Source: Endocr Connect. 2023 Dec 14;13(1):e230403. doi: 10.1530/EC-23-0403 (PMC10762563; doi:10.1530/EC-23-0403)

Suppl. Figure 1

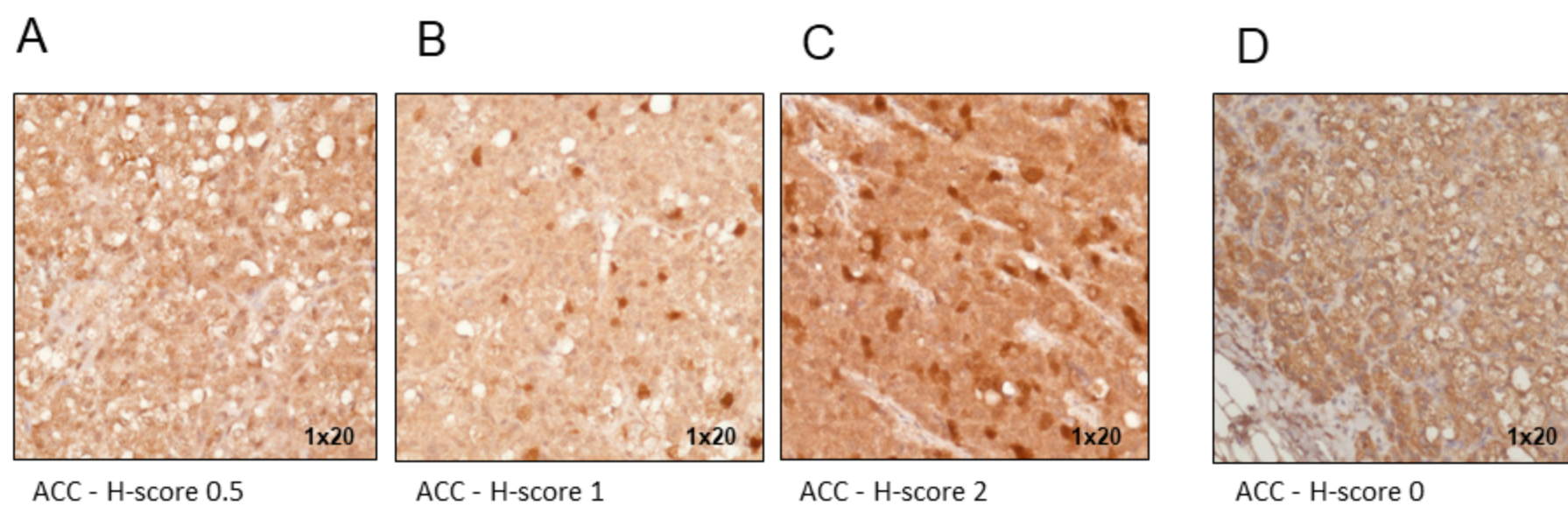

Supplement: Suppl. Figure 1. Examples of representative nuclear PLK1 immunostaining. A) – B) Adrenocortical carcinoma (ACC) with weak PLK1 staining; C) Adrenocortical carcinoma (ACC) with strong PLK1 staining; D) Normal adrenal gland used as negative control. Magnification 20x. H-score calculated as described i [file supplementary_figure_1.pdf]

Suppl. Figure 3

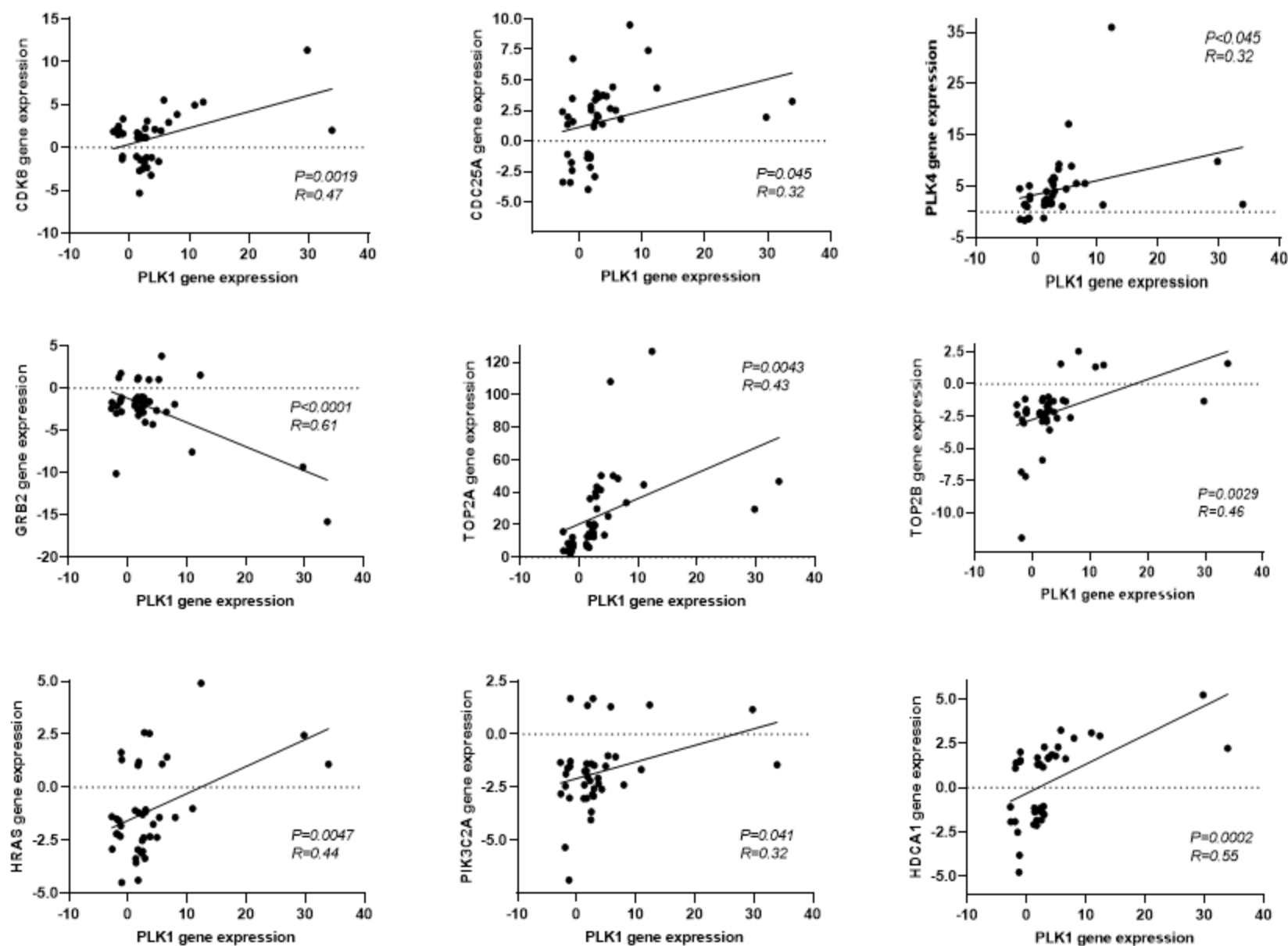

Supplement: Suppl. Figure 3. Relationship of mRNA expression levels between PLK1 and other known anti-cancer drug targets. Data taken from our previously published cohort of 40 paraffin-embedded ACC samples (9). Shown are expression levels for significant correlations, i.e. negatively with AKT2, BIRC5, CDC25A,  [file supplementary_figure_3.pdf]

Suppl. Figure 4

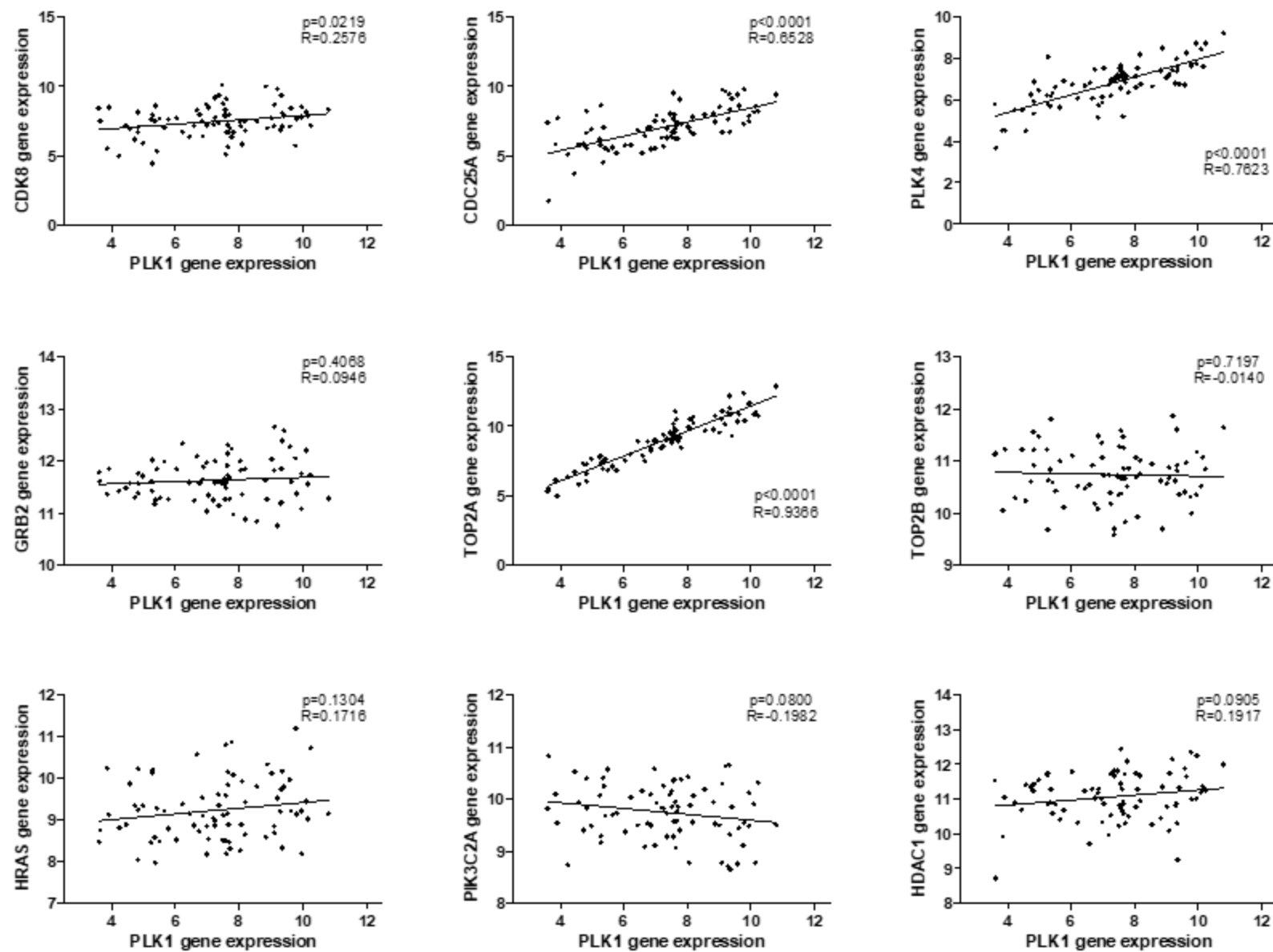

Supplement: Suppl. Figure 4. Most significant correlations with PLK1 gene expression in our previously published dataset of 40 paraffin-embedded ACC samples (9) were analysed in the TCGA ACC RNAseq dataset. Of note, PLK1 expression also positively correlated with CDK8, CDC25A, PLK4, and TOP2A. [file supplementary_figure_4.pdf]
