# Supplementary material for: PLK1 inhibitors as a new targeted treatment for adrenocortical carcinoma
Source: Endocr Connect. 2023 Dec 14;13(1):e230403. doi: 10.1530/EC-23-0403 (PMC10762563; doi:10.1530/EC-23-0403)
Supplement: Suppl. Figure 2. Schematic summarising molecular alterations observed in the four investigated ACC cell lines (NCI-H295R, MUC-1, CU-ACC1 and CU-ACC2). These include the response to treatment with PLK1 inhibitors rigosertib and poloxin (classified according to effects on cell proliferation), DNA alte [file supplementary_figure_2.pdf]

Suppl. Figure 2

ACC cell lines		NCI-H295R	MUC-1	CU-ACC1	CU-ACC2
**Response to treatment in vitro**					
Response to rigosertib		+++	+	-	+
Response to poloxin		++	+	-	++
**DNA alterations**					
	**Gene names**				
	ATRX	Essential splice site	Nonsense		
	CSFR1R	Missense			
	CTNNB1	Missense		Missense	
	KDR	Frameshift			
	MEN1		Missense		
	MSH2				Deletion
	TP53	Deletion	Frameshift		Missense

Legend: Red - nonsense mutation, blue - missense mutation, orange - frameshift/deletion, grey - essential splice site

Cell cycle related gene expression (fold-change)

Pathway/family	Gene names	NCI-H295R	MUC-1	CU-ACC1	CU-ACC2
Aurora kinase	AURKA	5.74	3.95	9.54	0.31
	BIRC5	19.38	21.16	10.28	7.35
Cyclin dependent kinase	CDC25A	10.98	5.99	14.95	14.3
	CDK1	179.94	81.08	217.27	99.46
	CDK2	9.05	5.26	5.49	7.16
	CDK4	1.15	0.37	1.78	2.23
	CDK5	1.65	0.49	2.72	0.66
	CDK8	1.13	0.64	7.72	0.62
	CDK9	0.55	1.12	2.53	0.31
Ras family	HRAS	0.56	0.36	1.91	0.48
	KRAS	2.81	1.22	2.03	2.43
	NRAS	1.02	0.45	1.16	2.24
IGF system	IGF1R	0.14	0.32	0.17	1.13
	IGF2	20.15	0.17	136.08	2.78
	PIK3CA	0.47	0.3	1.3	0.05
PLK family	PLK1	4.68	2.21	9.23	4.12
	PLK2	5.15	3.73	0.56	3.04
	PLK4	11.75	18.02	14.52	14.51
	TERT	0.33	0.62	2.02	0.46
Toposiomerase	TOP2A	133.1	68.58	144.17	122.59
